# Why the Single-N Design Should Be the Default in Affective Neuroscience

**DOI:** 10.1007/s42761-023-00182-5

**Published:** 2023-03-21

**Authors:** Håkan Fischer, Mats E. Nilsson, Natalie C. Ebner

**Affiliations:** 1 Department of Psychology, Stockholm University, 106 91 Stockholm, Sweden; 2 Stockholm University Brain Imaging Center (SUBIC), 106 91 Stockholm, Sweden; 3 Department of Psychology, University of Florida, Gainesville, FL 32611 USA; 4 Institute of Aging, University of Florida, Gainesville, FL 32611 USA; 5 Center for Cognitive Aging and Memory, Department of Clinical and Health Psychology, University of Florida, Gainesville, FL 32611 USA; 6 Florida Institute for Cybersecurity Research, University of Florida, Gainesville, FL 32610-0165 USA

**Keywords:** Psychophysics approach, Brain imaging, Methods, Emotion

## Abstract

Many studies in affective neuroscience rely on statistical procedures designed to estimate population averages and base their main conclusions on group averages. However, the obvious unit of analysis in affective neuroscience is the individual, not the group, because emotions are individual phenomena that typically vary across individuals. Conclusions based on group averages may therefore be misleading or wrong, if interpreted as statements about emotions of an individual, or meaningless, if interpreted as statements about the group, which has no emotions. We therefore advocate the Single-N design as the default strategy in research on emotions, testing one or several individuals extensively with the primary purpose of obtaining results at the individual level. In neuroscience, the equivalent to the Single-N design is deep imaging, the emerging trend of extensive measurements of activity in single brains. Apart from the fact that individuals react differently to emotional stimuli, they also vary in shape and size of their brains. Group-based analysis of brain imaging data therefore refers to an “average brain” that was activated in a way that may not be representative of the physiology of any of the tested individual brains, nor of how these brains responded to the experimental stimuli. Deep imaging avoids such group-averaging artifacts by simply focusing on the individual brain. This methodological shift toward individual analysis has already opened new research areas in fields like vision science. Inspired by this, we call for a corresponding shift in affective neuroscience, away from group averages, and toward experimental designs targeting the individual.

Many studies in affective neuroscience rely on statistical procedures designed to estimate population averages and base their main conclusions on group averages (Alvarsson et al., 2010; Ebner et al., 2013).^1^ The group, however, has no emotions and what is true of a group average may not apply to all or even most of the individuals in the group, so inferences from group averages to psychological phenomena and their neural substrates in individuals are only justified with additional evidence that individual differences are negligible, and strong evidence is required since people tend to differ on most psychological phenomena. In this article, we argue that the default research design in affective neuroscience should be the Single-N design (e.g., Barlow & Nock, 2009; Grice et al., 2017; Normand, 2016; Smith & Little, 2018), in which one individual is tested repeatedly on each experimental condition, including baseline conditions, with the goal to obtain sufficient data to draw firm conclusions about how the individual reacts to the experimental conditions*.*^2^

What can go wrong with group averages? Consider a neuroimaging experiment in which 30 individuals were tested under two experimental conditions, and the group average of the brain-activity contrast between the two experimental conditions was used to support the claim that “the stimulus manipulation had a moderate effect on neural activity in a brain area known to regulate behavioral response to this manipulation.” This would be perfectly true if all participants reacted in the same way to the stimulus manipulation. However, the claim is equally consistent with a scenario in which the manipulation had no effect on, say, a third of the participants, a moderate effect on another third, and a strong effect on the remaining third. In this scenario, it would be misleading to claim that the stimulus manipulation had a moderate effect as this was true only for a minority of the tested participants. Or, even worse, maybe the effect was negligible in about half of the participants but evoked a strong response in the other half, then the claim of a moderate effect would simply be false. Revising to “…moderate effect *at the group level*…” is no solution; it just makes the statement meaningless, as the group has no neural system that could be affected, moderately or otherwise.

But wouldn’t individual differences be seen in the data, as the average brain-activity contrast in the example above was derived from individual contrasts? Well, that depends on the design of the study, and specifically on how much data was gathered from each individual. If the study design involves few repetitions per experimental condition and individual, as is typical in studies using a within-subject design, it would be hard to distinguish true effects from random error at the individual level, and thereby hard to assess how representative the group average is of each individual’s data. Note that a common selling point for the within-subject design is that it may increase the precision of estimated effects at the *group level*, by removing variability due to individual differences at baseline. This may be true but irrelevant for studies in affective neuroscience targeting the individual. The defining characteristic of the Single-N design (Smith & Little, 2018), in which one individual is tested repeatedly on each experimental condition^3^, and which we argue here should be the default research design in affective neuroscience, is not that it is a within-subject design, but that its primary aim is to assess how a single individual, or each of several individuals, responds to the experimental manipulation. To achieve this goal requires extensive testing, to separate true effects from random errors at the individual level. Note that extensive testing rarely is restricted to repeated measurements of responses to a few experimental condition, but would typically also involve (a) systematic variation of stimuli to estimate outcomes based on their functional relationship with a specific stimuli dimension, (b) psychophysical methods to derive thresholds expressed in well-defined physical units from hundreds of responses from a single individual, and (c) clever designs to avoid habituation due to repeated presentation of stimuli that, by itself, could affect behavior as well as brain activation in regions like the amygdala (Fischer et al., 2003; Plichta et al., 2014).

A knee-jerk reaction to the Single-N design is that it lacks external validity as you cannot generalize to the population from only one or a few tested individuals. But generalization comes second, first you need to demonstrate that your results reflect real phenomena and are not artifacts of averaging across individuals. To demonstrate this, the Single-N design is needed, unless you can be sure that individual differences are negligible, which seems a very strong assumption for anything involving human emotions. Of course, with unlimited resources, we could apply the Single-N design to large samples of individuals drawn randomly from the target population(s) to describe the phenomenon at both the individual and the group level. However, resources are not unlimited, and we typically have to choose between testing a few individuals extensively or testing a large number of individuals sparsely. We would typically prefer the former as it allows us to describe the results at the level where emotions and their neural substrates occur, the individual.

There are of course research problems in affective neuroscience for which the Single-N design is inappropriate. For example, the research aim may be to assess the prevalence of an emotional disorder in the population, or to assess the relative risk of an emotional disorder in a specific population compared to the general population. These are questions that can only be answered with data from large samples from the target population(s). For other research problems, the Single-N design may be the ideal in principle, but carry-over effects may make it practically impossible. For example, the surprise triggered by an unexpected stimulus may not be triggered on a second exposure to the same stimulus. Carry-over effects can often be minimized via clever experimental design, but when not possible, researchers may have to settle with group-level analyses. However, these limitations notwithstanding, the Single-N should be the design that comes first to mind when designing a study in affective neuroscience, because of the intrinsic individual nature of human emotions. Designs targeting group averages and group comparisons are the second best, and should be resorted to only if the Single-N design is not feasible or cannot answer the research question asked, or if the research question requires the study of a psychological phenotype across two analysis levels, the individual and the group level. For example, when the goal is to determine which nodes in a neural network that fall within the general group pattern for each subject versus which nodes are unique, that are specific to an individual. This information would advance understanding of similarities versus differences between the neural mechanisms of a psychological phenotype across these two analysis levels.

But is not averaging within an individual as problematic as averaging across individuals? For example, an individual may react strongly in some trials and not at all in others, with an average suggesting a moderate reaction, although a moderate reaction never occurred. Many studies using the Single-N design indeed only report the average response of an individual, and are therefore open to this objection. But, of course, the remedy is simple, just test extensively enough to be able to assess the within-subject distributions (and not only averages) of responses to experimental conditions. And maybe this is one future direction for research in affective neuroscience, accepting the fact that emotions not only vary across individuals but also within individuals. This perspective is in line with findings that emotion categories are populations of instances, and should therefore be studied as such (Siegel et al., 2018; Mau et al., 2021).

Deep neuroimaging (Gratton & Braga, 2021) is the neuroscience research equivalent of the Single-N design (Smith & Little, 2018), in focusing on a single brain and many repeated experimental trials*.* The deep neuroimaging approach contrasts the trend in neuroimaging research over the past two decades that favored the collection of larger and larger sample sizes towards the goal of enhancing replicability in cognitive neuroscience (Turner et al., 2018) or enabled the study of brain-genome relationships (e.g., UK Biobank) and interindividual differences in the neural underpinnings of psychological outcomes (e.g., Human Connectome Project). Using deep neuroimaging to study human brain function is currently unexplored territory with potential to advance understanding of the human brain at work in the field of affective neuroscience.

Besides the point already raised that emotions are individual experiences and should be investigated as such, deep neuroimaging makes it possible to avoid interindividual variability in brain physiology; if that is not the target of the study as for example in the study of how interindividual differences in brain physiology are associated with interindividual differences in cognitive function in adult aging (MacDonald et al., 2009; Bäckman et al., 2006). Deep neuroimaging also makes it possible to avoid effects of interindividual variability in brain structure on the phenomenon under study. Although most human brains are structurally similar on a broad level, there are individual differences in brain shape and size. To do group analysis in neuroimaging studies, each brain needs to be transformed (or warped) to a standard space to have the same size, shape, and dimensions so that corresponding pixels in different brains can be compared. By normalizing individual brains to a common space, the spatial localization of brain signals in individual brains is less clear. Even more, when these individual brains are warped, the average location of the brain signal might not be representative of any single subject in the group. Even though, in the normalization process, each individual’s structural and functional images have been transformed to match the general shape and large anatomical features of the group-derived template, there still remain variations in how smaller anatomical regions align. To account for this problem in the group-average analysis, individual images are smoothed (i.e., the brain activation is “smeared out”). By doing this, there is more overlap between clusters of signal regardless of remaining interindividual anatomical differences, and therefore greater likelihood of detecting a significant effect. This preprocessing step of smoothing, however, influences the spatial localization of activity in individual brains in addition to the effect normalization has on spatial localization. In deep neuroimaging, normalization of brain data is not needed because the individual brain is only compared to itself over repeated assessments and smoothing could for the same reason be kept to a minimum. This, in turn, will enable researchers to differentiate nearby brain activations and structural changes, by using the full potential of the constantly improving spatial resolution in the development of modern brain imaging scanners.

A recent paper (Cheng et al., 2022) proposes that for fMRI, large numbers of trials are as important for statistical estimation as large numbers of subjects. From a statistical estimation perspective, more is better, both in terms of the number of subjects and the number of trials. But from a deep neuroimaging perspective, one could argue that a larger number of trials in single individuals is preferred over fewer trials in a larger number of subjects because the focus should be on individual and not group processes.

In conclusion, deep neuroimaging is at the forefront of a methodological paradigm shift by measuring each individual extensively with the goal to best represent individual brains (i.e., to separate true effects from measurement error at the individual level). This approach has great potential for significant conceptual advancement in affective neuroscience. Deep neuroimaging has already opened new research areas in fields like vision science (Naseralis et al., 2021), cognitive control (Smith et al., 2021), and brain plasticity (Newbold & Dosenbach, 2021), and can do so also in affective neuroscience. We argue that a brain model of affective processing in humans should be based on high-rate repeated sampling of large stimulus variation within individuals. From a deep neuroscience perspective, understanding of the neural basis of affective processing in single subjects will allow for understanding activity in any one human brain. This rationale underlies our call for a methodological shift in affective neuroscience, from research designs targeting group averages based on small sets of responses from each of a large number of individuals, toward designs targeting individual data, based on extensive sets of responses from one or several individuals. Ideally, the Single-N approach should be run in multiple subjects resulting in multiple independent studies. This will also allow for the investigation of small neuroimaging samples using a within-person group-based approach, preferable with a more longitudinal design and some kind of intervention, to increase measurement reliability and effect sizes (Marek et al., 2022). Study designs that combine both deep neuroimaging and a more traditional mean-based within-group approach in the same study protocol (i.e., a subgroup of individuals is scanned extensively over time) are very powerful regarding the robustness of their prediction while at the same time allowing for some generalizability across subjects.

## Footnotes

1 We cite ourselves, not to boost our citation indices, but to avoid pointing fingers at others and to be transparent with having adhered to the group-averaging approach that we criticize in this paper.

2 That being said, it should be acknowledged that a combination of the Single-N approach and the traditional group-based approach can be beneficial for many research questions in affective neuroscience, for example, questions that require both high precision in terms of spatial resolution, which deep neuroimaging in single subject will provide (Gratton & Braga, 2021), and more generalizable average patterns of brain response for the psychological phenotype under study. It should furthermore be made clear that what we are arguing for regarding the use of the Single-N design in affective neuroscience also applies to cognitive neuroscience as well as other fields of science (e.g., social neuroscience).

3 Often more than one individual is tested in Single-N studies, representing multiple replications of the same experiment on different individuals (Smith & Little, 2018); and results should be presented separately for each individual.

## References

[CR1] Alvarsson JJ, Wiens S, Nilsson ME (2010). Stress recovery during exposure to nature sound and environmental noise. International Journal of Environmental Research and Public Health.

[CR2] Bäckman L, Nyberg L, Lindenberger U, Li SC, Farde L (2006). The correlative triad among aging, dopamine, and cognition: Current status and future prospects. Neuroscience and Biobehavioral Reviews.

[CR3] Barlow D, Nock MK (2009). Why can’t we be more idiographic in our research?. Perspectives on Psychological Science.

[CR4] Cheng G, Pine DS, Brotman MA, Smith AR, Cox RW, Taylor PA (2022). Hyperbolic trade-off: The importance of balancing trial and subject sample sizes in neuroimaging. NeuroImage.

[CR5] Ebner NC, Johnson MR, Rieckmann A, Durbin KA, Johnson MK, Fischer H (2013). Processing own-age vs. other-age faces: Neuro-behavioral correlates and effects of emotion. NeuroImage.

[CR6] Fischer, H., Wright, C.I., Whalen, P.J., McInerney, S.C., Shin, L.M., & Rauch, S.L. (2003). Brain habituation during repeated exposure to fearful and neutral faces: A functional MRI study. *Brain Research Bulletin*, *59*(5), 387–392. https://www.ncbi.nlm.nih.gov/pubmed/12507690. Accessed 30 Jan 2003 10.1016/s0361-9230(02)00940-1 12507690

[CR7] Gratton C, Braga RM (2021). Editorial overview: Deep imaging of the individual brain: Past, practice, and promise. Current Opinion in Behavioral Science.

[CR8] Grice J, Barrett P, Cota L, Felix C, Taylor Z, Garner S, Medellin E, Vest A (2017). Four bad habits of modern psychologists. Behavioral Science.

[CR9] MacDonald SW, Li SC, Bäckman L (2009). Neural underpinnings of within-person variability in cognitive functioning. Psychology and Aging.

[CR10] Marek S, Tervo-Clemmens B, Calabro FJ, Montez DF, Kay BP, Hatoum AS (2022). Reproducible brain- wide association studies require thousands of individuals. Nature.

[CR11] Mau, L.T., Hoemann, K., Lyons, S.H. et al. (2021). Professional actors demonstrate variability, not stereotypical expressions, when portraying emotional states in photographs. *Nature Communication, 12*. p. 5037. 10.1038/s41467-021-25352-6 10.1038/s41467-021-25352-6 PMC8376986 34413313

[CR12] Naseralis T, Allen E, Kay K (2021). Extensive sampling for complete models of individual brains. Current Opinion in Behavioral Science.

[CR13] Newbold DJ, Dosenbach NUF (2021). Tracking plasticity in individual human brains. Current Opinion in Behavioral Science.

[CR14] Normand MP (2016). Less is more: Psychologists can learn more by studying fewer people. Frontiers in Psychology.

[CR15] Plichta MM, Grimm O, Morgen K, Mier D, Sauer C, Haddad L (2014). Amygdala habituation: A reliable fMRI phenotype. NeuroImage.

[CR16] Siegel EH, Sands MK, Van den Noortgate W, Condon P, Chang Y, Dy J (2018). Emotion fingerprints or emotion populations? A meta-analytic investigation of autonomic features of emotion categories. Psychological Bulletin.

[CR17] Smith PL, Little DR (2018). Small in beautiful: In defense of the small-N design. Psychonomic Bulletin and Review.

[CR18] Smith DM, Perez DC, Porter A, Dworetsky A, Gratton C (2021). Light through the fog: Using precision fMRI data to disentangle the neural substrates of cognitive control. Current Opinion in Behavioral Science.

[CR19] Turner BO, Paul EJ, Miller MB, Barbey AK (2018). Small sample sizes reduce the replicability of task-based fMRI studies. Communications Biology.

